# ID 175 - Monitoramento do Horizonte Tecnológico de Potenciais Vacinas para a Profilaxia do Vírus Zika e Chikungunya

**DOI:** 10.5327/2237-9622.2025.v34s1.175

**Published:** 2025-11-25

**Authors:** Munique Gonçalves Guimarães, Viviane Del Lama Cardoso Salas, Ana Carolina de Freitas Lopes, Luciene Fontes Schluckebier Bonan

**Keywords:** vacina, monitoramento do horizonte tecnológico, vírus Zika, vírus chikungunya

## Abstract

**Introdução:**

As arboviroses são doenças virais, transmitidas pela picada do mosquito infectado, que coloca em risco quase 4 bilhões de pessoas no mundo, de acordo com a Organização Mundial da Saúde. O Brasil, por ser um País com zonas tropicais e subtropicais, torna-se um ambiente favorável para a proliferação desses insetos, como dengue, Zika e chikungunya. Atualmente, não há tratamento específico para as arboviroses; os analgésicos e antipiréticos são indicados de forma paliativa para amenizar a dor e a febre. Assim, tendo em vista a implementação da vacina contra o mosquito por meio do Programa Nacional de Imunizações (PNI) e o cenário epidemiológico, este trabalho tem como objetivo buscar no horizonte tecnológico, tecnologias novas e emergentes que poderá imunizar a população contra o vírus Zika e chikungunya.

**Método:**

Foram realizadas buscas nas bases de dados ClinicalTrials.gov3 e Cortellis™4 no período de 26/2/2024 a 13/3/2024, utilizando-se os seguintes termos na estratégia de busca: "Zika Virus infection" and "Chikungunya virus infection". Foram considerados estudos clínicos de vacinas de fases 1, 2 e 3; em andamento ou completo até cinco anos. Para a situação regulatória das tecnologias selecionadas, foram consultados os sítios eletrônicos: Agência Nacional de Vigilância Sanitária (Anvisa), European Medicines Agency (EMA) e U.S. Food and Drug Administration (FDA) com indicação clínica de até cinco anos, nos dias 18 e 19 de março de 2024.

**Resultados:**

O Monitoramento do Horizonte Tecnológico (MHT) identificou oito vacinas para o vírus Zika, sendo: mRNA-1893; ChAdOx1 Zika; Zika Virus Purified Inactivated Vaccine (ZPIV); VRC-ZKADNA090-00-VP; Purified Inactivated Zika Virus Vaccine (PIZV); rZIKV/D4Δ30-713; MV-ZIKA-RSP e ZIKV-IG e nenhuma vacina possui registro sanitário na Agência Nacional de Vigilância Sanitária (Anvisa), EMA ou FDA. Para o vírus chikungunya foram apontadas seis vacinas, a saber: BBV87, MV-CHIK, ChAdOx1 Chik, mRNA-1388, VLA1553 e PXVX-0317 e somente a vacina (VLA1553 -IXCHIQ®) já possui registro sanitário no FDA, e pedido de registro em andamento na Anvisa e na EMA.

**Conclusão:**

Os imunizantes em estágio mais avançado para o vírus Zika são a mRNA-1893 e a VRC-ZKADNA090-00-VP. O mRNA-1893 é uma vacina de RNA mensageiro (mRNA) que utiliza o peptídeo do vírus da encefalite japonesa com a tecnologia V1GL8. Já o VRC-ZKADNA090-00-VP é uma vacina de DNA do vírus Zika composta por um único plasmídeo de DNA circular fechado que codifica as proteínas da membrana percursora M e envelope da cepa H/PF/2013 do vírus Zika. Este estudo está sendo conduzido no Brasil e em oito países e seu ensaio clínico está registrado na Anvisa em fase 2 e possui cooperação estrangeira. Para o vírus chickugunya, destaca-se as vacinas MV-CHIK e VLA1553, com vetor de vírus vivo atenuado. No ensaio clínico concluído de fase 3 foi constatado segurança, tolerabilidade e imunogenicidade adequados. A PXVX0317, vacina de partículas semelhantes ao vírus da chikungunya, já apresenta ensaios clínicos de fase 3. No Brasil, a vacina VLA1553 está em desenvolvimento pelo Instituto Butantan em parceria com a farmacêutica Valneva Áustria GmbH em sete centros de pesquisa: Belo Horizonte, Boa Vista, Campo Grande, Fortaleza, Laranjeiras, Manaus, Recife, Salvador, São José do Rio Preto e São Paulo. Em suma, ainda não há vacina disponível para prevenção da infecção pelo vírus chikungunya e pelo vírus Zika (CHIKV) no Brasil, porém há estudos em desenvolvimento.

